# RNF213 isoform 2 restricts Zika virus through antiviral signaling and viral protein degradation

**DOI:** 10.1016/j.isci.2026.116284

**Published:** 2026-06-05

**Authors:** Xiaoyu Yang, Teng Chen, Bin Ren, Jinqiu Liu, Zhen Xu, Jingyi Wang, Yiran Shi, Lihua Liu, Lili Li, Genhong Cheng, Dongming Zhou, Qi Chen, Shulong Zu

**Affiliations:** 1 Department of Pathogen Biology, School of Basic Medical Sciences, Tianjin Medical University, Tianjin 300070, China; 2 Key Laboratory of Jilin Province for Zoonoses Prevention and Control, State Key Laboratory of Pathogen and Biosecurity, Academy of Military Medical Sciences, Changchun 130122, China; 3 Senior Department of Neurosurgery, The First Medical Center of Chinese PLA General Hospital, Beijing 100853, China; 4 State Key Laboratory of Pathogen and Biosecurity, Academy of Military Medical Sciences, Beijing 100071, China; 5 Department of Toxicology and Health Inspection and Quarantine, School of Public Health, Tianjin Medical University, Tianjin 300070, China; 6 School of Basic Medicine, Gannan Medical University, Ganzhou 341000, China; 7 Guangzhou National Laboratory, Guangzhou 510005, China

**Keywords:** biochemistry, virology

## Abstract

Several RING finger (RNF) family proteins exert antiviral effects primarily by regulating host antiviral pathways. Moreover, the longer isoform of RNF213 can also directly target viral proteins to inhibit infection. However, the antiviral potential of the shorter isoform of RNF213 (RNF213 isoform 2) remains unexplored. Here, we report that RNF213 isoform 2 (hereafter referred to as RNF213) activates the retinoic acid-inducible gene I (RIG-I)-melanoma differentiation-associated gene 5 (MDA5) pathway and promotes proteasomal and lysosomal degradation of multiple Zika virus (ZIKV) proteins, including the capsid (C), envelope (E), nonstructural 3 (NS3), and nonstructural 4B (NS4B) proteins, to restrict ZIKV infection. Notably, we identified a 23-amino acid peptide (PR-23) derived from RNF213 that degrades ZIKV proteins and suppresses viral replication *in vitro*. In conclusion, our findings not only elucidate a dual-targeting antiviral mechanism of RNF213 against ZIKV but also define a minimal functional domain of this isoform that mediates antiviral activity *in vitro*.

## Highlights

• RNF213 isoform 2 activates the antiviral signaling to constrain ZIKV infection
• RNF213 isoform 2 restricts ZIKV replication by targeting viral proteins for degradation
• PR-23 from RNF213 isoform 2 inhibits ZIKV infection via viral protein degradation

## Graphical abstract

**Figure:**
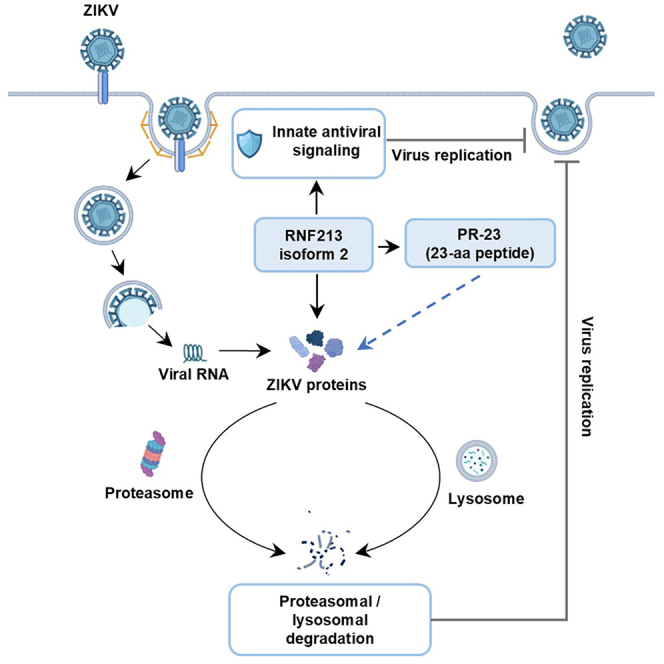

## Introduction

Zika virus (ZIKV), a member of the genus *Orthoflavivirus* within Flaviviridae family, was first isolated in 1947 from a rhesus macaque in the Zika Forest of Uganda.^1^ ZIKV infection typically presents as a self-limiting illness characterized by fever, headache, and maculopapular rash.^2^ During 2015–2016, an outbreak of ZIKV was firstly reported in Brazil, and by 2016, the epidemic had spread rapidly across the Americas.^3^ This epidemic revealed that ZIKV infection in pregnant women is associated with microcephaly in newborns, and the virus can also induce Guillain-Barré syndrome and other neurological sequelae in adults.^4^ In light of its teratogenic effects and pandemic potential, the World Health Organization (WHO) declared ZIKV a Public Health Emergency of International Concern (PHEIC) in February 2016. Despite extensive research efforts, no licensed antiviral therapies or vaccines are currently available, underscoring the urgent need for effective countermeasures against this pathogen.

ZIKV is an enveloped, positive-sense, single-stranded RNA (ssRNA) virus, with a ∼10.8 kb genome encoding three structural proteins: capsid (C), precursor membrane (prM), and envelope (E), as well as seven nonstructural (NS) proteins: NS1, NS2A, NS2B, NS3, NS4A, NS4B, and NS5.^5^ The structural proteins are responsible for viral particle assembly and pathogenicity, while the nonstructural proteins participate in multiple stages of the viral life cycle, such as viral replication, assembly, and hijacking of host pathways for immune evasion.^6^ There are two main genotypes of ZIKV (African and Asian), and the Asian genotype causes the majority of outbreaks.^7^

The RING finger (RNF) family represents the largest class of E3 ubiquitin ligases in humans, comprising over 340 members that regulate diverse cellular processes.^8^ Existing evidence shows that some RNF family E3 ubiquitin ligases exert antiviral effects primarily by regulating host antiviral pathways.^9^ For instance, RNF144A modulates STING ubiquitination to promote antiviral responses^10^; RNF214 is a critical component of the innate immune response and broadly restricts ssRNA viruses^11^; and RNF216 suppresses H5N1 avian influenza virus replication by modulating the retinoic acid-inducible gene I (RIG-I)-mediated antiviral signaling pathway in ducks.^12^ However, recent studies suggest that some RNFs, such as RNF213, can not only regulate host antiviral pathways^13^ but also have the potential to directly target the virus itself. It has been reported that RNF213 targets the replication and transcription activator (RTA) of herpesvirus, promoting its degradation through the proteasome-dependent pathway.^14^^,^^15^ The aforementioned studies suggest that RNF213 may possess broader antiviral potential.

RNF213 produces multiple isoforms through alternative splicing. Isoform 2 (1,063 amino acids) is shorter than the isoform 3 (5,207 amino acids) and shares identical 1,008 N-terminal residues with the isoform 3. Current research on the antiviral properties of RNF213 has predominantly focused on the longer isoform 3. However, its size complicates experimental manipulation and druggability. Given its small size and higher druggability potential, we investigated whether RNF213 isoform 2 possesses antiviral activity against ZIKV. In this study, we identified E3 ubiquitin protein ligase RNF213 isoform 2 (hereafter referred to as RNF213) as a host restriction factor that inhibits ZIKV infection. Mechanistically, RNF213 could upregulate the RIG-I- melanoma differentiation-associated gene 5 (MDA5) pathway, which is consistent with the antiviral mechanisms observed in other members of the RNF family. More importantly, we demonstrated that RNF213 mediates ZIKV protein degradation primarily via the proteasomal and lysosomal pathways, thereby effectively suppressing viral replication. This degradation activity represents a previously unrecognized function within the RNF family. Notably, we demonstrated that the truncations containing 55-amino acid (R55) and 23-amino acid (R23) fragments of RNF213 protein retain the capacity to degrade ZIKV proteins and suppress viral replication. Building on these findings, we designed a synthetic peptide based on the R23 sequence that recapitulated the antiviral activity of R23-expressing plasmid *in vitro*. Therefore, our results establish RNF213 as a host restriction factor against ZIKV infection and highlight the therapeutic potential of RNF213-derived peptides as broad-spectrum antiviral agents.

## Results

### RNF213 inhibits ZIKV replication *in vitro*

**Figure 1:**
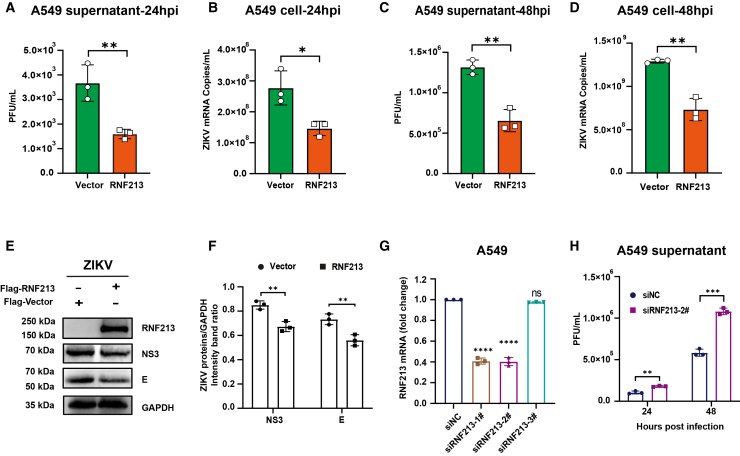
RNF213 inhibits ZIKV replication *in vitro* (A–F) A549 cells were transfected with the plasmid encoding Flag-tagged RNF213 or vector control. After 24 h, the cells were infected with ZIKV (MOI = 0.01). (A–D) At 24 and 48 hpi, the viral RNA loads in the cell supernatant and cells were quantified by plaque assay and RT-qPCR. (E) At 48 hpi, cell lysates were harvested and detected by western blot; GAPDH served as an internal control. (F) Densitometry analysis of data in (E). (G) A549 cells were transfected with negative control (NC) siRNA (siNC) and RNF213 siRNA (siRNF213). After 48 h, total RNA of the cells was extracted, and the mRNA levels of RNF213 were quantified by RT-qPCR. (H) A549 cells were transfected with siNC and siRNF213. After 24 h, the cells were infected with ZIKV (MOI = 0.01). At 24 and 48 hpi, the viral RNA loads in cell supernatant were determined by plaque assay. Data are expressed as the mean ± SD from three independent experiments (*n* = 3; ns, *p* > 0.05; ∗, *p* < 0.05; ∗∗, *p* < 0.01; ∗∗∗, *p* < 0.001; and ∗∗∗∗, *p* < 0.0001).

To evaluate the antiviral activity of RNF213 against ZIKV, A549 cells were transfected with RNF213-expressing plasmid followed by infection with ZIKV (MOI = 0.01). At 24 and 48 h post-infection (hpi), viral replication was measured by plaque assay and quantitative reverse transcription PCR (RT-qPCR). The results showed that RNF213 overexpression significantly reduced viral RNA loads in both cell supernatants and cells (Figures 1A–1D). In addition, western blot analysis was performed to detect the protein levels in ZIKV-infected A549 cells overexpressing RNF213 or control vector at 48 hpi; the result revealed that RNF213 overexpression also reduced ZIKV protein expressions (Figures 1E and 1F). Furthermore, we employed small interfering RNA (siRNA) technology to perform RNF213 knockdown assays, and the result demonstrated that siRNA-mediated silencing of RNF213 effectively reduced the mRNA expression level of RNF213 in A549 cells (Figure 1G). Subsequently, A549 cells that were transfected with siRNF213 were infected with ZIKV, and the viral replication was measured using plaque assay. The results showed that the knockdown of endogenous RNF213 could enhance viral RNA loads in cell supernatants (Figure 1H). Taken together, these results showed that RNF213 could inhibit ZIKV replication *in vitro*.

### RNF213 positively regulates the RIG-I-MDA5 pathway

**Figure 2:**
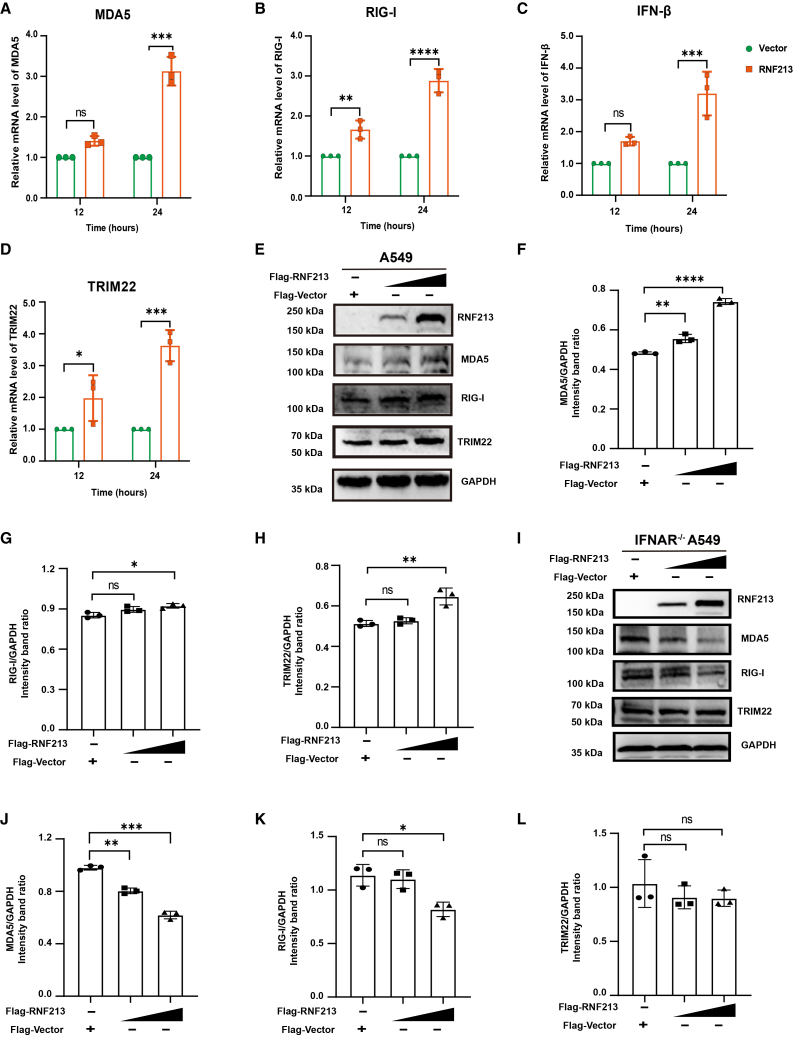
RNF213 positively regulates the RIG-I-MDA5 pathway (A–D) A549 cells were transfected with the plasmid encoding Flag-tagged RNF213 or vector control. After 12 and 24 h, total RNA of the cells was extracted, and the mRNA levels of *MDA5* (A), *RIG-I* (B), *IFN-β* (C), and *TRIM22* (D) were quantified by RT-qPCR. (E–H) A549 cells were transfected with the indicated amount of plasmid encoding Flag-tagged RNF213 or vector control. The levels of MDA5, RIG-I, or TRIM22 in A549 cells were measured using western blot assay. GAPDH served as an internal control. (F and H) Densitometry analysis of data in (E). (I–L) The experiment in (E) was repeated using IFNAR-knockout A549 cells. (J–L) Densitometry analysis of data in (I). Data are expressed as the mean ± SD from three independent experiments (*n* = 3; ns, *p* > 0.05; ∗, *p* < 0.05; ∗∗, *p* < 0.01; ∗∗∗, *p* < 0.001; ∗∗∗∗, *p* < 0.0001).

RNF family proteins are critical in regulating the RIG-I-MDA5 and downstream signaling pathways.^9^ To investigate the regulatory effect of RNF213 on the RIG-I-MDA5 pathway, A549 cells were transfected with an RNF213-expressing plasmid or a vector control in a 12-well plate. At 12 and 24 h post-transfection, the total RNA of cell lysates was extracted for RT-qPCR. The results of RT-qPCR showed that the mRNA levels of *MDA5*, *RIG-I*, *IFN-β*, interferon-stimulated gene (ISG), and *TRIM22* were significantly upregulated in RNF213 overexpression cells compared with negative control cells (Figures 2A–2D). Furthermore, A549 cells were transfected with increasing amounts of the RNF213-expressing plasmid or a vector control. At 36 h post-transfection, the cell lysates were harvested for western blot analysis. The results revealed that RNF213 overexpression significantly upregulated the protein levels of MDA5, RIG-I, and TRIM22 in A549 cells (Figures 2E–2H). To dissect the underlying mechanism by which RNF213 elevates the expression of RIG-I-like receptors (RLR), we subsequently performed an enzyme-linked immunosorbent assay (ELISA) to characterize the role of RNF213 in type I interferon (IFN-I) production in A549 cells. The result demonstrated that RNF213 was functionally implicated in the induction of IFN-I (Figure S1). To further validate the dependency of this regulatory effect on IFN-I signaling, we repeated the experiment depicted in Figure 2E, using interferon receptor (IFNAR)-knockout A549 cells. Notably, RNF213 failed to enhance the expression of the aforementioned proteins in IFNAR-knockout cells (Figures 2I–2L). Collectively, these findings supported the conclusion that RNF213 serves as a positive regulator of the RIG-I-MDA5 signaling pathway, and this regulatory role is mediated by increased IFN-I production.

### RNF213 targets ZIKV C, E, NS3, and NS4B proteins for degradation

**Figure 3:**
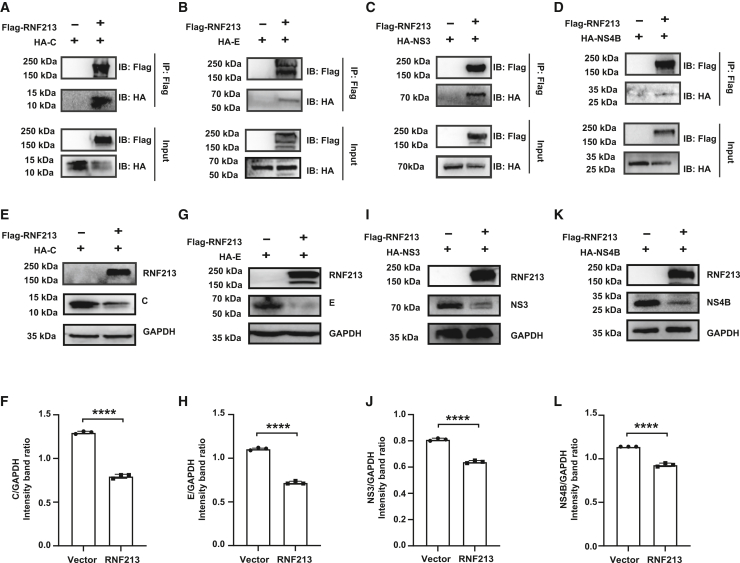
RNF213 targets ZIKV C, E, NS3, and NS4B proteins for degradation (A–D) HEK293T cells were transfected with the plasmid encoding Flag-tagged RNF213, together with plasmids encoding HA-tagged ZIKV C, E, NS3, or NS4B protein. Cell lysates were then immunoprecipitated (IP) with anti-Flag beads, and immunoblotting (IB) analysis was performed with an anti-HA antibody to detect ZIKV proteins. (E–L) Western blot analysis of lysates from HEK293T cells transfected with the plasmid encoding Flag-tagged RNF213 or vector control, together with plasmids encoding HA-tagged ZIKV C, E, NS3, or NS4B protein. GAPDH served as an internal control. Data are expressed as the mean ± SD from three independent experiments (*n* = 3; ∗∗∗∗, *p* < 0.0001).

To further elucidate the mechanism underlying RNF213-mediated ZIKV restriction, a co-immunoprecipitation (coIP) assay was performed to identify viral proteins that interact with RNF213. Our analysis revealed specific interactions between RNF213 and multiple ZIKV structural (prM, C, and E) and nonstructural (NS1, NS3, and NS4B) proteins (Figures 3A–3D, S2A, and S2B), while no interactions were detected with NS4A and NS5 (Figures S2C and S2D). Furthermore, we investigated the effect of RNF213 on the stability of viral proteins. For this purpose, HEK293T cells were co-transfected with RNF213-expressing and ZIKV protein-expressing plasmids. Western blot results showed that RNF213 overexpression reduced the levels of C, E, NS3, NS4A, NS4B, and NS5 (Figures 3E–3L and S2I–S2L) in a dose-dependent manner (Figures S3A, S3C, and S3E–S3H). In contrast, prM and NS1 levels were not affected by the presence of RNF213 (Figures S2E–S2H, S3B, and S3D). Taken together, these results suggested that RNF213 interacts with C, E, NS3, and NS4B and promotes the degradation of these viral proteins.

### RNF213 mediates ZIKV protein degradation via the proteasomal and lysosomal pathways

**Figure 4:**
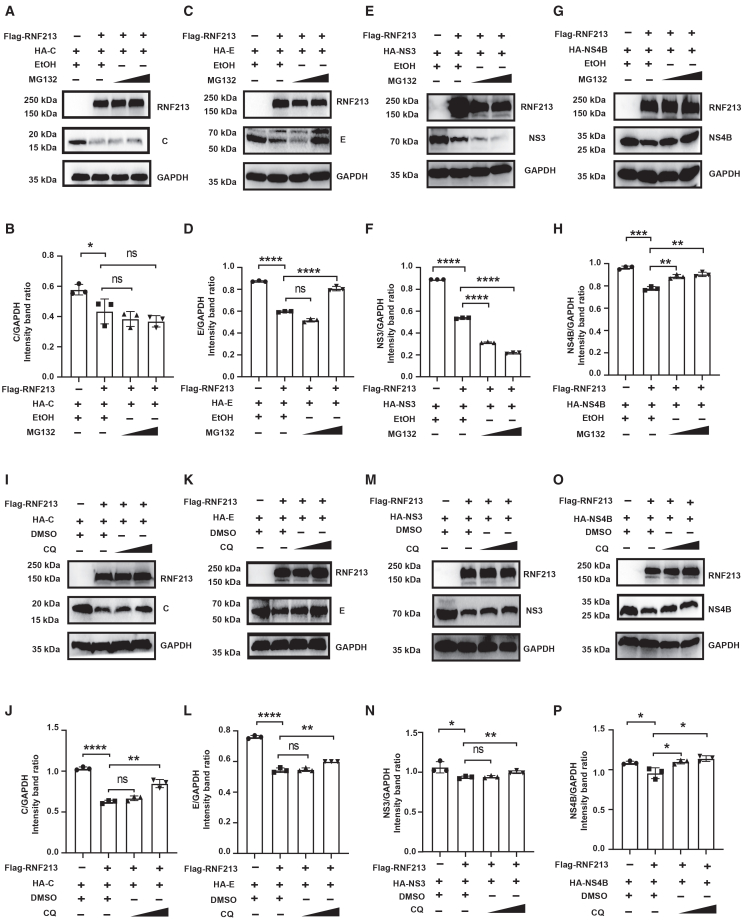
RNF213 mediates ZIKV protein degradation via multiple pathways (A–P) HEK293T cells were transfected with the plasmid encoding Flag-tagged RNF213, together with plasmids encoding HA-tagged ZIKV proteins. MG132 (10 and 20 μM) (A–H) or chloroquine (CQ, 20 and 30 μM) (I–P) was added at 24 h post-transfection, and the cells were harvested after 6 h of incubation; ethanol (EtoH) and dimethyl sulfoxide (DMSO) were used as negative controls. The levels of ZIKV proteins were evaluated by the western blot assay. GAPDH served as an internal control. Data are expressed as the mean ± SD from three independent experiments (*n* = 3; ns, *p* > 0.05; ∗, *p* < 0.05; ∗∗, *p* < 0.01; ∗∗∗, *p* < 0.001; ∗∗∗∗, *p* < 0.0001).

To explore the pathways of degradation through which RNF213 mediates the degradation of viral proteins, the proteasome inhibitor MG132 or the lysosome inhibitor chloroquine (CQ) was added to the cells previously transfected with RNF213-expressing and ZIKV protein-expressing plasmids. Western blot analysis revealed that RNF213-mediated reduction in the C and NS3 protein levels persisted under MG132 treatment, while the levels of E and NS4B proteins were restored (Figures 4A–4H). To further explore whether RNF213 modulates E and NS4B ubiquitination, GFP-tagged E or His-tagged NS4B and HA-tagged WT or K48 ubiquitin, along with Flag-tagged RNF213 plasmid or a vector control were co-transfected into HKE293T cells. Indeed, our IP assays revealed a significant elevation in the ubiquitination levels of both E and NS4B proteins upon RNF213 expression (Figure S4). In contrast, the C, E, NS3, and NS4B levels were all effectively rescued by CQ treatment (Figures 4I–4P). These results suggested that RNF213 orchestrates viral protein degradation through both the proteasomal and lysosomal pathways in a target-specific manner.

### Truncations of RNF213 degrade ZIKV proteins and inhibit viral replication

**Figure 5:**
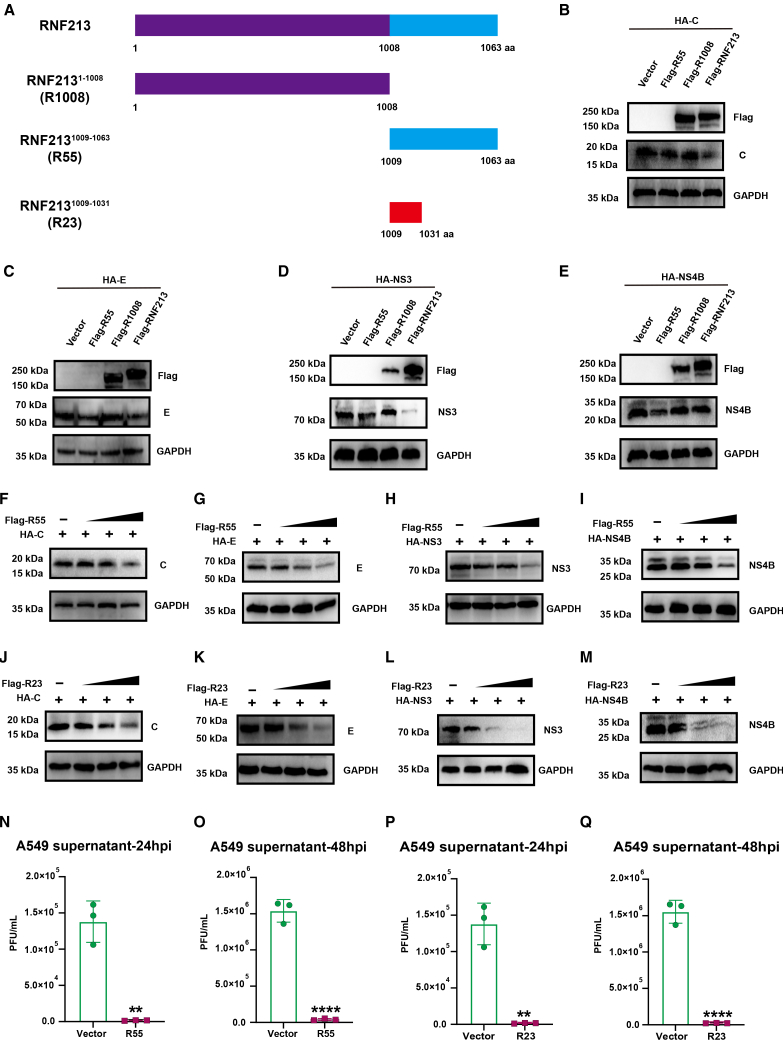
Truncations of RNF213 degrade ZIKV proteins and inhibit viral replication (A) Schematic of RNF213 and its truncations. (B–E) HEK293T cells were transfected with plasmids encoding Flag-tagged RNF213 and its truncations or vector control, together with plasmids encoding HA-tagged ZIKV proteins. The levels of ZIKV proteins were evaluated by the western blot assay. The protein level of Flag-R55-expressing plasmid was not detected, likely due to its small size. GAPDH served as an internal control. (F–M) HEK293T cells were transfected with the indicated amount of plasmids encoding Flag-tagged R55 or R23, together with plasmids encoding HA-tagged ZIKV C, E, NS3, or NS4B protein. Cell lysates were collected for western blot analysis. The protein levels of Flag-R55- and Flag-R23-expressing plasmids were not detected, likely due to their small size. GAPDH served as an internal control. (N–Q) A549 cells were transfected with or without plasmids encoding Flag-tagged RNF213 truncations (R55 and R23), and then infected with ZIKV (MOI = 0.01). Viral replication was measured by plaque assay. Data are expressed as the mean ± SD from three independent experiments (*n* = 3; ∗∗, *p* < 0.005; ∗∗∗∗, *p* < 0.0001).

Given that the N-terminal 1,008 amino acids of RNF213 share complete sequence identity with those of isoform 3, and the N-terminal 1,008 amino acids belong to the N arm of RNF213 isoform 3, which is not a special function domain, we generated three RNF213 truncations to map the functional domain responsible for viral protein degradation (Figure 5A). Co-expression studies in HEK293T cells revealed that both full-length RNF213 and the 55-amino acid truncation (R55), but not the 1,008-amino acid truncation (R1008), significantly reduced the levels of ZIKV C, E, NS3, and NS4B proteins (Figures 5B–5E and S5). Furthermore, R55 could degrade ZIKV proteins in a dose-dependent manner (Figures 5F–5I). In addition, R55 was tested in the cells infected with ZIKV. Similar to the viral protein degradation experiments, R55 overexpression could suppress ZIKV replication in A549 cells (Figures 5N and 5O). Remarkably, a minimal 23-amino acid truncation (R23) derived from R55 retained the capacity to degrade ZIKV proteins in a dose-dependent manner and significantly reduced viral titers in cell supernatants (Figures 5J–5M, 5P, and 5Q). To determine whether R23, like full-length RNF213, upregulates the RIG-I-MDA5 pathway, A549 cells were transfected with an R23-expressing plasmid or a vector control in a 12-well plate. At 24 h post-transfection, total RNA of the cell lysates was extracted for RT-qPCR. The results of RT-qPCR showed that the mRNA levels of *RIG-I*, *IFN-β*, and *TRIM2*2, but not *MDA5*, were upregulated in R23 overexpression cells (Figures S6A–S6D). Western blot results showed that R23 could also enhance the protein expression of TRIM22 (Figures S6E and S6F). Mechanistically, R23 degraded ZIKV proteins primarily via the lysosomal pathway, which was consistent with those in RNF213 overexpression experiments (Figure S7). These findings suggested that the truncations of RNF213 have the potential to act as therapeutic peptides to inhibit ZIKV replication.

### RNF213-derived antiviral peptide suppresses ZIKV replication via viral protein degradation

**Figure 6:**
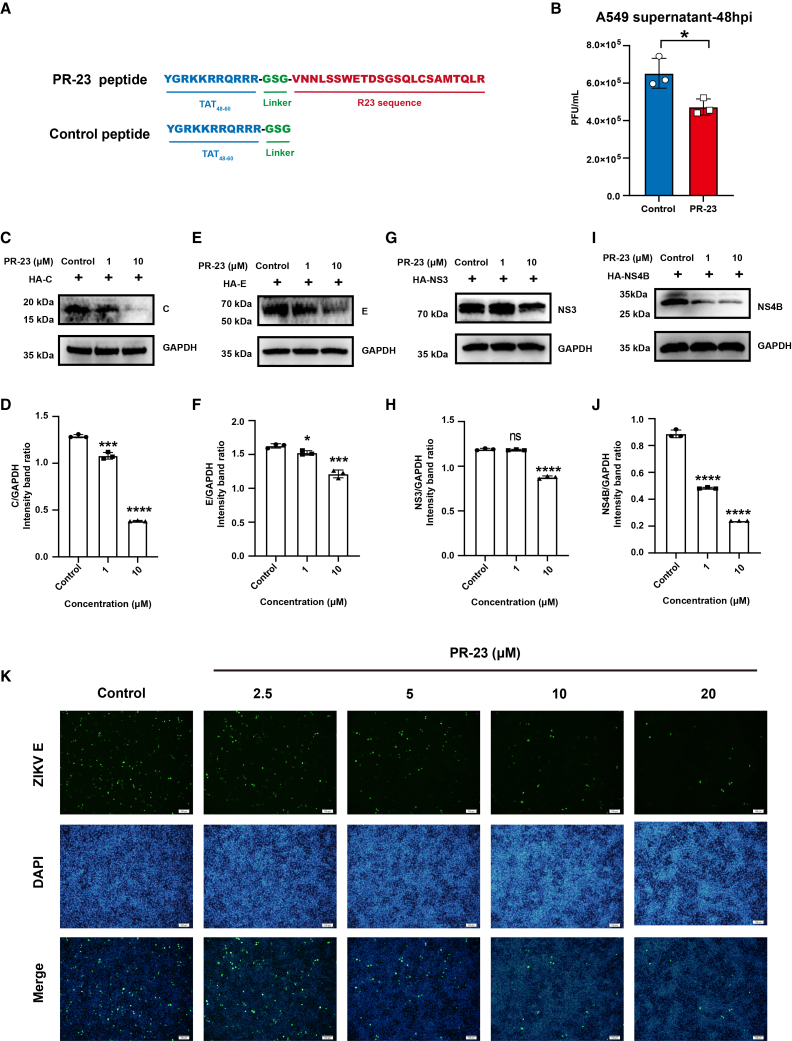
Peptide from RNF213 truncation degrades ZIKV proteins and inhibits viral replication (A) The sequence of peptide from RNF213 truncation (PR-23) and control peptide. (B) A549 cells were infected with ZIKV (MOI = 0.01) and then treated with PR-23 (10 μM). Viral replication of cell supernatant was measured by plaque assay at 48 hpi. (C–J) HEK293T cells were treated with the indicated amount of PR-23 for 24 h, and then transfected with plasmids encoding HA-tagged ZIKV proteins. After 36 h, cell lysates were harvested and detected by western blot assay. GAPDH served as an internal control. (K) Vero cells were treated with the indicated dose of PR-23 for 6 h and transfected with the plasmid encoding GFP-tagged ZIKV E protein. After 36 h, the cells were fixed and subjected to immunofluorescence assay. The nucleus was stained with DAPI (blue). Images were obtained using a laser confocal microscope. Scale bars, 100 μm. Data are expressed as the mean ± SD from three independent experiments (*n* = 3; ns, *p* > 0.05; ∗, *p* < 0.05; ∗∗∗, *p* < 0.001; ∗∗∗∗, *p* < 0.0001).

Building on the observed antiviral activity of R23, we designed a 23-amino acid synthetic peptide (PR-23) by incorporating the R23 sequence with an N-terminal human immunodeficiency virus-1 (HIV-1) TAT_47–57_ cell-penetrating peptide (CPP) (YGRKKRRQRRR) and a GSG linker, and a control peptide that contained only the CPP and GSG linker (Figure 6A). We next tested the cytotoxicity of PR-23 in Vero cells by using CCK-8 assay, and the result showed that PR-23 had no significant cytotoxicity at concentrations up to 133 μM (Figure S8). To evaluate the inhibitory activity of PR-23 against ZIKV infection, we performed plaque assay using ZIKV-infected A549 cells. The result showed that PR-23 treatment led to a marginal decrease in viral RNA accumulation (Figure 6B). Importantly, HEK293T cells were treated with the indicated dose of PR-23 and then transfected with ZIKV protein-expressing plasmids. Western blot and immunofluorescence assay results showed that the PR-23 treatment could significantly reduce ZIKV protein expressions (Figures 6C–6K and S9). These lines of evidence indicated that PR-23, as a peptide-based antiviral agent, could be a promising antiviral strategy against ZIKV infection.

## Discussion

In this study, we first identified RNF213 and its truncations as new ZIKV antagonists and revealed the mechanism by which RNF213 inhibits ZIKV infection. We then explored the possibility of using an RNF213 truncation as a potential antiviral candidate. Based on the sequence of RNF213 truncation, we designed a peptide, PR-23, which effectively degraded ZIKV proteins and inhibited ZIKV RNA replication *in vitro*. These findings demonstrate that this peptide exerted antiviral activity against ZIKV infection in our experimental models.

RIG-I and MDA5 have been proven to play a crucial role in controlling RNA viral infection and activating the signaling pathway of IFN production.^16^ IFN stimulates the expression of hundreds of ISGs, some of which may restrict different types of viral infection.^17^ Previous reports have shown that RNF proteins are responsible for regulating the RIG-I-MDA5 and downstream signaling pathways.^9^ RNF135 interacts with RIG-I, catalyzing the K63-linked ubiquitination of its C-terminal region, thereby facilitating the activation of RIG-I.^18^^,^^19^^,^^20^ A gene-knockout study demonstrated that RNF135 plays a critical role in RIG-I activation and subsequent antiviral IFN responses against RNA virus infection *in vivo.*^21^ Specifically, during severe acute respiratory syndrome coronavirus 2 (SARS-CoV-2) infection, RNF135 deficiency significantly reduced IFN-I production and markedly increased viral replication. These findings suggest that RNF135 could be a potential therapeutic target for treating coronavirus disease 2019 (COVID-19).^9^ Furthermore, RNF194 (also known as MEX3C), an RNA-binding protein, preferentially co-localizes with viral RNA and RIG-I. This spatial proximity enables it to catalyze K63-linked polyubiquitination on the N-terminal caspase activation and recruitment domains (CARDs) of RIG-I, thereby potentiating RIG-I-mediated antiviral signaling.^22^ RNF218 (also known as MUL1/MAPL), a mitochondrial localized E3 ligase, SUMOylates RIG-I in an energy- and temperature-dependent manner, thereby facilitating its activation. This modification exposes the two CARDs, promoting the assembly of the mitochondrial antiviral signaling (MAVS) signaling complex.^23^ Additionally, RNF54 is essential for IFN responses, which restrict acute viral replication and enhance the systemic clearance of murine norovirus (MNoV).^24^

RNF213 is unique in that it is the only known protein to exhibit a dual enzymatic function, combining ATPases associated with diverse cellular activities (AAA+) ATPase activity with ubiquitin ligase capability.^25^ While initial research efforts predominantly focused on elucidating the function of RNF213 in Moyamoya disease (MMD). Echizenya et al. subsequently identified its therapeutic potential for alleviating severe headache symptoms in patients with enterovirus-induced hand, foot, and mouth disease.^26^ The unique structural feature of RNF213 indicates its potential for multifunctional roles. Notably, RNF213 can catalyze diverse ubiquitin chains, including M1, K11, K48, and K63, depending on the specific pathogens involved.^14^^,^^27^^,^^28^ Emerging evidence has established that RNF213 plays an important role in cerebrovascular disorders triggered by viral infections, thereby providing initial insights into the mechanistic connection between RNF213 and virus.^26^ Comparative analysis revealed that mice deficient in RNF213 were significantly more susceptible to Rift Valley fever virus (RVFV) infection. Conversely, mice overexpressing RNF213 *in vivo* demonstrated markedly improved viral resistance, with attenuated infection severity compared with wild-type controls.^29^ Furthermore, the experimental infection with pathogenic avian influenza strains induced a substantial upregulation of RNF213 expression in avian species.^30^ RNF213 acts as an E3 ubiquitin ligase, catalyzing K48-linked polyubiquitination of the viral RTA protein and inducing its degradation by proteasome. Downregulation of RTA subsequently attenuates the initiation of downstream viral replication and transcriptional processes. Notably, emerging evidence suggests that RNF213 may facilitate viral clearance through T cell-mediated mechanisms, thereby contributing to its broad-spectrum antiviral activity.^31^ These findings provide evidence that RNF213 acts as an antiviral factor, which plays a protective role in host defense against ZIKV.

The size of RNF213 isoform 3 (5,207 amino acids) presents a significant challenge for experimental manipulation, making it difficult to study the protein. In contrast, RNF213 consists of 1,063 amino acids and has a significantly shorter C- terminal region than isoform 3, which make it easier to understand the structure and function of RNF213. Consequently, we used RNF213 in this study and found that it could upregulate the RIG-I-MDA5 pathway and degrade ZIKV proteins to inhibit viral infection. We propose that RNF213 functions as an important inhibitory mechanism against ZIKV replication.

The growing challenge of antiviral resistance, coupled with the ongoing threat to global public health posed by emerging viral pathogens such as Ebola, ZIKV, and SARS-CoV-2, highlights the urgent need for continued and intensive research into novel antiviral therapeutics.^32^ In addition to conventional small-molecule antivirals and biologics, antimicrobial peptides (AMPs) have recently emerged as a promising class of antiviral therapeutics.^33^^,^^34^ Studies have shown that antiviral peptides (AVPs) exhibit various mechanisms of action, such as disrupting the viral envelope and interfering with multiple stages of the viral life cycle, including inhibiting initial attachment and suppressing viral egress from infected host cells.^35^^,^^36^^,^^37^ Furthermore, rational design approaches have emerged as a powerful strategy for developing optimized AMPs with enhanced potency and specificity, facilitating the identification of lead compounds with superior therapeutic selectivity. Structural analysis reveals that most AVPs are relatively short polypeptides comprising fewer than 100-amino acid residues on average, with a mean length of approximately 31 residues. In this study, we designed and constructed a peptide, PR-23, derived from RNF213 sequence. The PR-23 peptide shares the same sequence as the R23 overexpression plasmid, and both promote the degradation of Zika viral proteins in cells. However, the antiviral activity of PR-23 remains weak *in vitro*, and its antiviral efficacy has not yet been evaluated *in vivo* and requires further in-depth investigation.

In summary, our study demonstrates that RNF213 exerts antiviral activity against ZIKV by upregulating the RIG-I-MDA5 pathway, increasing IFN and ISG levels, and facilitating the degradation of viral proteins by multiple pathways—representing a previously unrecognized antiviral mechanism. Furthermore, we propose that PR-23 may be a promising candidate for controlling ZIKV infection.

### Limitations of the study

This study has several limitations that warrant consideration. First, the antiviral activity of the RNF213-derived peptide PR-23 is strictly a proof-of-concept finding, and its *in vivo* efficacy, safety profile, and potential for clinical translation have not been evaluated and require further systematic investigation. Second, direct evidence supporting the degradation of viral proteins by RNF213 is well established only for a subset of ZIKV proteins. We have not validated whether RNF213 can directly interact with or degrade other ZIKV-encoded components, and the precise molecular mechanisms underlying its interaction with the limited subset of viral proteins remain to be fully elucidated. Third, a critical unresolved issue is the lack of quantitative analysis to dissect the relative contribution of the RIG-I-MDA5 pathway (and subsequent IFN signaling) activation versus viral protein turnover to the overall antiviral effect of RNF213. We have demonstrated that both mechanisms are operative, but their respective roles—including which pathway predominates in mediating antiviral activity—have not been clarified, which limits our understanding of the core mode of action of RNF213.

Finally, the broad-spectrum antiviral activity of RNF213 against other flaviviruses was not directly tested in this study. As such, the generalizability of our findings to the broader *Flavivirus* genus remains limited, and further experiments are needed to determine whether the observed antiviral effects are specific to ZIKV or extend to other related viruses.

## Resource availability

### Lead contact

Requests for further information, resources, and reagents should be directed to and will be fulfilled by the lead contact, Shulong Zu (zushulong@tmu.edu.cn).

### Materials availability

All unique/stable reagents generated in this study will be available from the lead contact with a completed material(s) transfer agreement.

### Date and code availability

• Any data presented in this study will be shared by the lead contact upon request.
• This paper does not report original code.
• Any additional information required to reanalyze the data reported in this paper is available from the lead contact upon request.

## Acknowledgments

This project was financially supported by the Fundation of 10.13039/501100015976State Key Laboratory of Pathogen and Biosecurity of China (SKLPBS2441) and the National Natural Science Foundation of China (32441098). L.L. is supported by 10.13039/501100004608Natural Science Foundation of Jiangsu Province (BK20241810).

## Author contributions

S.Z., Q.C., G.C., and D.Z. conceived the project; S.Z. and Q.C. designed and supervised the study and wrote the paper; X.Y., T.C., B.R., and J.L. performed most of the experiments; Z.X., J.W., Y.S. L. Liu, and L. Li. contributed specific experiments and data analysis. All authors contributed to the writing and editing of the manuscript.

## Declaration of interests

The authors declare that they have no competing interests.

## Declaration of generative AI and AI-assisted technologies in the writing process

During the preparation of this work, the authors used ChatGPT in order to check and refine the language. After using this tool, the authors reviewed and edited the content as needed and take full responsibility for the content of the publication.

## STAR★Methods

### Key resources table

**Table:** 

REAGENT or RESOURCE	SOURCE	IDENTIFIER
**Antibodies**		

Rabbit anti-HA-peroxidase	Roche	Cat# 12013819001; RRID: AB_390917
Mouse anti-Flag-Tag monoclonal antibody (HRP Conjugated)	Beyotime	Cat# AF2855; RRID: AB_3674126
Rabbit anti-MDA5 polyclonal antibody	Beyotime	Cat# AF7164; RRID: AB_3751255
Rabbit anti-RIG-I polyclonal antibody	Beyotime	Cat# AF7890; RRID: AB_3751256
Mouse anti-GAPDH monoclonal antibody	Beyotime	Cat# AF2819; RRID: AB_3751257
Rabbit anti-TRIM22 polyclonal antibody	Novus Biologicals	Cat# NBP1-81795; RRID: AB_11012511
Rabbit anti-Zika virus Envelope polyclonal antibody	GeneTex	Cat# GTX133326; RRID: AB_2886931
Rabbit anti-Zika virus NS3 polyclonal antibody	GeneTex	Cat# GTX133309; RRID: AB_2756864
Mouse anti-GFP monoclonal antibody	EASYBIO	Cat# BE2070; RRID: AB_3751258
Mouse anti-His monoclonal antibody	EASYBIO	Cat# BE2017; RRID: AB_3751259

**Bacterial and virus strains**		

ZIKV	Department of Virology, State Key Laboratory of Pathogen and Biosecurity, Beijing Institute of Microbiology and Epidemiology, China	N/A

**Chemicals, peptides, and recombinant proteins**		

PR-23	Synthesized at Genscript, China	N/A
Control peptide	Synthesized at Genscript, China	N/A
MG132	abcam	ab141003
Chloroquine	MedChemExpress	HY-17589A

**Critical commercial assays**		

Human IFN-β ELISA kit	Solarbio	SEKH-0410
Cell counting kit-8	Beyotime	C0037
LipofectamineTM 3000 Transfection Reagent	Thermo Fisher Scientific	2041107

**Experimental models: Cell lines**		

HEK293T	ATCC	ACS-4500
A549	ATCC	CCL-185
Vero	ATCC	CCL-81
IFNAR KO A549	This paper	N/A

**Oligonucleotides**		

See Table S1	N/A	N/A

**Recombinant DNA**		

Flag-tagged RNF213 plasmid	This paper	N/A
Flag-tagged RNF213 truncation plasmids	This paper	N/A
HA-tagged ZIKV expression plasmids	This paper	N/A
GFP-tagged ZIKV E plasmid	This paper	N/A
His-tagged ZIKV NS4B plasmid	This paper	N/A
HA-tagged ubiquitin plasmids	This paper	N/A

**Software and algorithms**		

GraphPad prism 9.0	Graphpad	https://www.graphpad.com/
ImageJ	ImageJ	https://imagej.net/ij/

### Experimental model and study participant details

#### Virus and cell lines

ZIKV strain (FSS13025, GenBank: JN860885) has been described in our previous work.^38^ Human embryonic kidney (HEK293T), human lung adenocarcinoma (A549), and African green monkey kidney (Vero) cells were purchased from the American Type Culture Collection (ATCC), IFNAR-knockout A549 cells were kindly provided by Prof. Genhong Cheng (Guangzhou National Laboratory). All the cells were cultured in Dulbecco’s modified Eagle medium (DMEM) supplemented with 10% fetal bovine serum (FBS), penicillin (100unit/mL), and streptomycin (100μg/mL) at 37 °C with 5% CO_2_. The cell lines were not further authenticated beyond the supplier’s characterization. All cells were routinely tested to be free of mycoplasma contamination.

### Method details

#### Plasmids

Flag-tagged RNF213 was preserved by our laboratory. Flag-tagged RNF213 truncation plasmids and HA-tagged ZIKV expression plasmids were constructed by using standard molecular cloning techniques and verified by sequencing. ZIKV E gene and NS4B gene were cloned into the pEGFP-C1 and pcDNA3.1-His expression vector, respectively, using standard molecular cloning techniques and verified by sequencing. HA-tagged ubiquitin plasmids were preserved by our laboratory.

#### Peptide synthesis

Peptide PR-23 (YGRKKRRQRRRGSGVNNLSSWETDSGSQLCSAMTQLR) and control peptide (YGRKKRRQRRRGSG) were synthesized at Genscript.

#### Small interference RNA synthesis

Three small interference RNAs (siRNAs) targeting human E3 ubiquitin protein ligase RNF213 isoform 2 and a negative control (NC) siRNA were designed and synthesized by OBiO. Primer sequences are listed in Table S1.

#### Immunoprecipitation and immunoblotting

HEK293T cells were transfected with the indicated plasmids. After transfection, the total protein from cells was extracted using Cell lysis buffer for Western and IP (Beyotime) containing PMSF (Solarbio). An aliquot of the extracts was immunoblotted with the indicated antibodies. The remaining extracts were immunoprecipitated using Sepharose beads (Beyotime) bound to anti-Flag antibody at 4 °C overnight. The Sepharose beads were washed three times with wash buffer, then proteins were eluted by heating the beads to 98 °C in 1×SDS–polyacrylamide gel electrophoresis loading buffer (Solarbio). The eluate and remaining whole cell extracts were analyzed by immunoblotting with the indicated antibodies. Immunoblotting was carried out by standard procedures as usual.

#### Detection of human interferon-β (IFN-β)

A549 cells were transfected with RNF213 expressing plasmid or control vector plasmid. At 24 and 48 h post-transfection, the cell supernatants were collected to detect the production of IFN-β. Human IFN-β in cell culture supernatant was quantified by human IFN-β ELISA kit (SEKH-0410, Solarbio) following the manufacturer’s protocol. Briefly, cell supernatants were centrifuged (1000×g, 4°C), aliquoted and stored at −20 °C. Kit and cell supernatants were equilibrated at room temperature, standards were serially diluted. After adding standards/cell supernatants to pre-coated microplates, incubation, washing and substrate reaction were performed sequentially. OD values (450/630 nm) were measured, and IFN-β concentration was calculated by four-parameter fitting standard curve, with dilution factor correction for diluted samples.

#### RNA preparation and real-time PCR

Total RNA from cells or cell supernatants was extracted with the PureLink RNA Extraction kit (Thermo Fisher Scientific). Viral RNA copies were measured by RT-qPCR with the One Step PrimeScript RT-PCR kit (Takara). ZIKV primers and TaqMan probes were described previously. SYBR qPCR mix (Novoprotein) was used to quantify the mRNA level of the genes. All primers used in this study are listed in Table S1.

#### Immunofluorescence staining

Vero cells were treated with the indicated doses of PR-23 for 6 h, and transfected with GFP-tagged ZIKV-E. 36 h after transfection, the cells were fixed with 4% paraformaldehyde at room temperature for 20 min. The cells were washed three times with 1×PBS. Nuclei were stained with DAPI (Solarbio). Finally, the images were captured using a fluorescence microscope.

#### Cytotoxicity assay

The cytotoxicity of PR-23 in Vero cells was determined by CCK-8. Briefly, Vero cells were treated with different doses of either compound or with control in triplicate. After 3 days of incubation at 37 °C, cytotoxicity assay was performed according to the manufacturer’s protocols. After adjusting the absorbance for background (medium) and comparing to untreated controls (untreated cell medium), the cytotoxic concentration was calculated using the GraphPad Prism 7.01 software.

### Quantification and statistical analysis

All data were analyzed using Prism software (GraphPad). Statistical evaluation was performed by unpaired two-tailed Student’s *t* test. Data are presented as means ± SD, and *p* values are indicated by ns *p* > 0.05, ∗*p* < 0.05, ∗∗*p* < 0.01, ∗∗∗*p* < 0.001, and ∗∗∗∗*p* < 0.0001. For Western blot data, representative data from at least three independent experiments are shown. All cellular experiments were repeated at least three times.
