# Supplementary material for: RNF213 isoform 2 restricts Zika virus through antiviral signaling and viral protein degradation
Source: iScience. 2026 Jun 5;29(6):116284. doi: 10.1016/j.isci.2026.116284 (PMC13266186; doi:10.1016/j.isci.2026.116284)
Supplement: Document S1. Figures S1–S9 and Table S1 [file mmc1.pdf]

iScience, Volume *29*

Supplemental information

RNF213 isoform 2 restricts Zika virus through antiviral signaling and viral protein degradation

Xiaoyu Yang, Teng Chen, Bin Ren, Jinqiu Liu, Zhen Xu, Jingyi Wang, Yiran Shi, Lihua Liu, Lili Li, Genhong Cheng, Dongming Zhou, Qi Chen, and Shulong Zu

Supplemental Figures

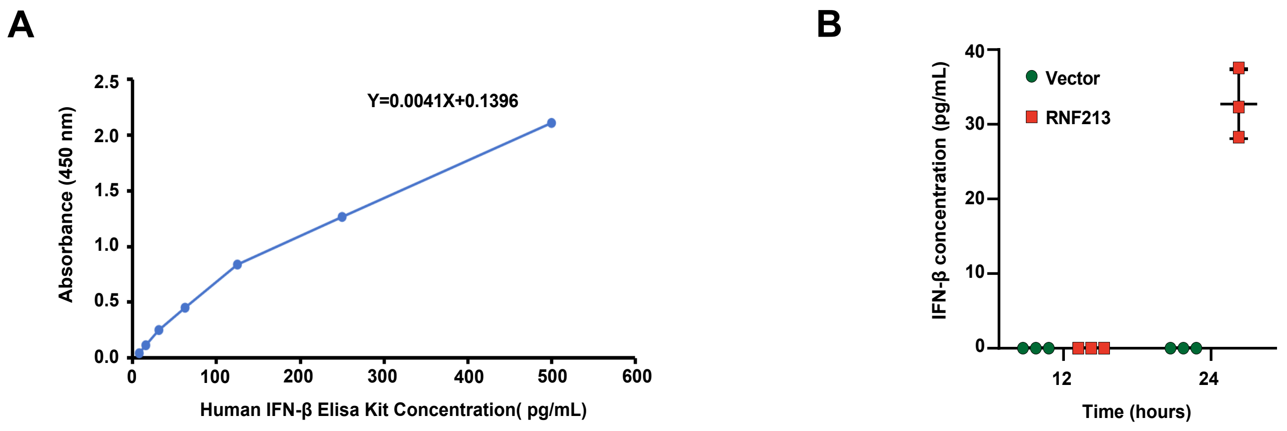

Figure S1. RNF213 overexpression induces IFN-β production in A549 cells.
(A-B) A549 cells were transfected with the plasmid encoding Flag-tagged RNF213 or vector control. At 24 and 48 hours post-transfection, the cell supernatant were collected for detecting IFN-β production by human IFN-β ELISA kit. (A) A standard curve was generated using the standard provided in the kit. (B) IFN-β concentration was calculated based on the established standard curve.

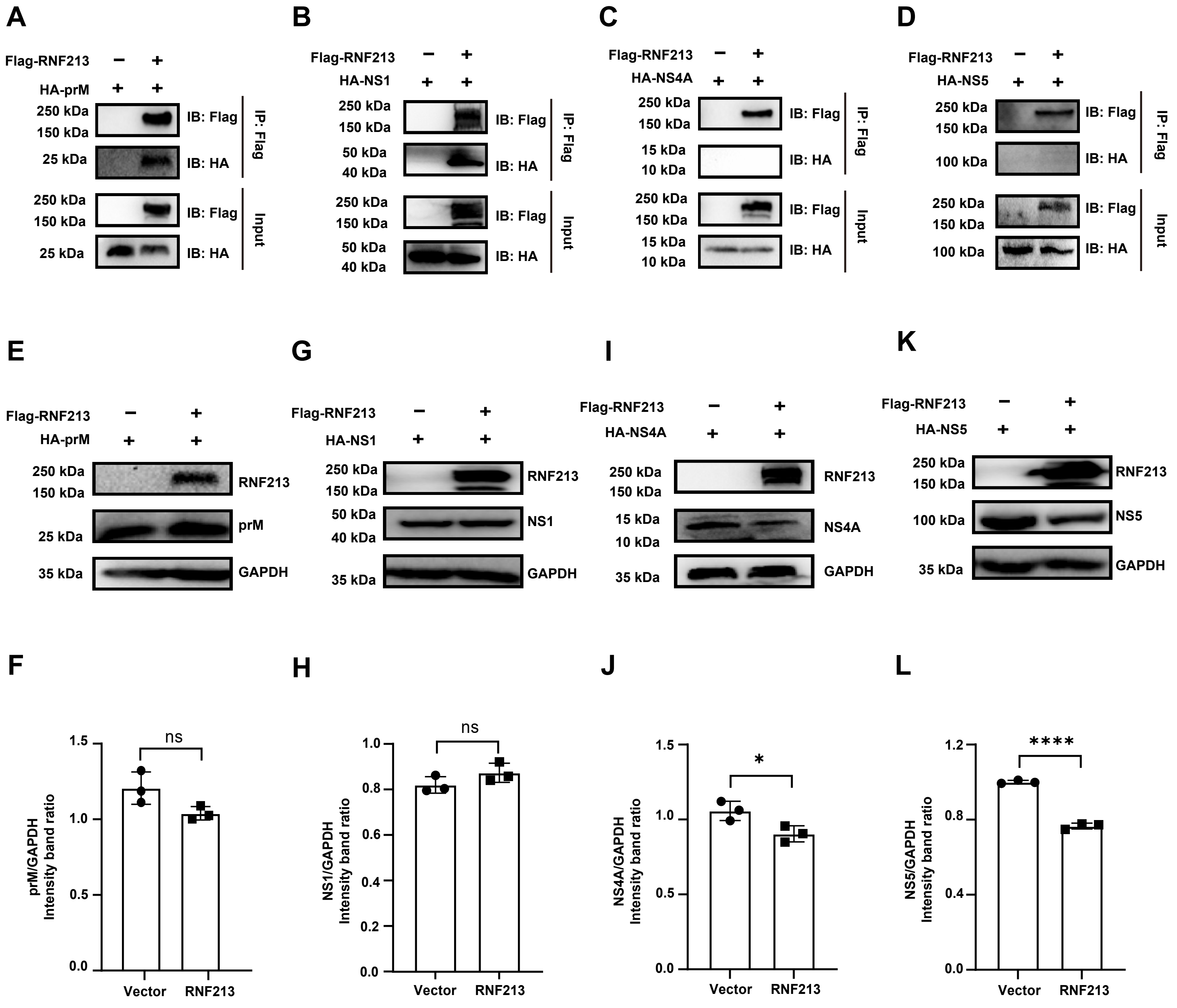

Figure S2. The interaction and degradation of RNF213 with other ZIKV proteins.

(A-D) HEK293T cells were transfected with plasmid encoding Flag-tagged RNF213, together with plasmids encoding HA-tagged ZIKV prM, NS1, NS4A or NS5 protein. Cell lysates were then immunoprecipitated (IP) with anti-Flag beads, and immunoblotting (IB) analysis was performed with an anti-HA antibody to detect ZIKV proteins. (E-L) Western blot analysis of lysates from HEK293T cells transfected with plasmid encoding Flag–tagged RNF213 or vector control, together with plasmids encoding HA-tagged ZIKV prM, NS1, NS4A or NS5 protein. GAPDH served as an internal control. Data are expressed as mean ± SD from three independent experiments (n = 3, ns $P > 0.05$, * $P < 0.05$, **** $P < 0.0001$).

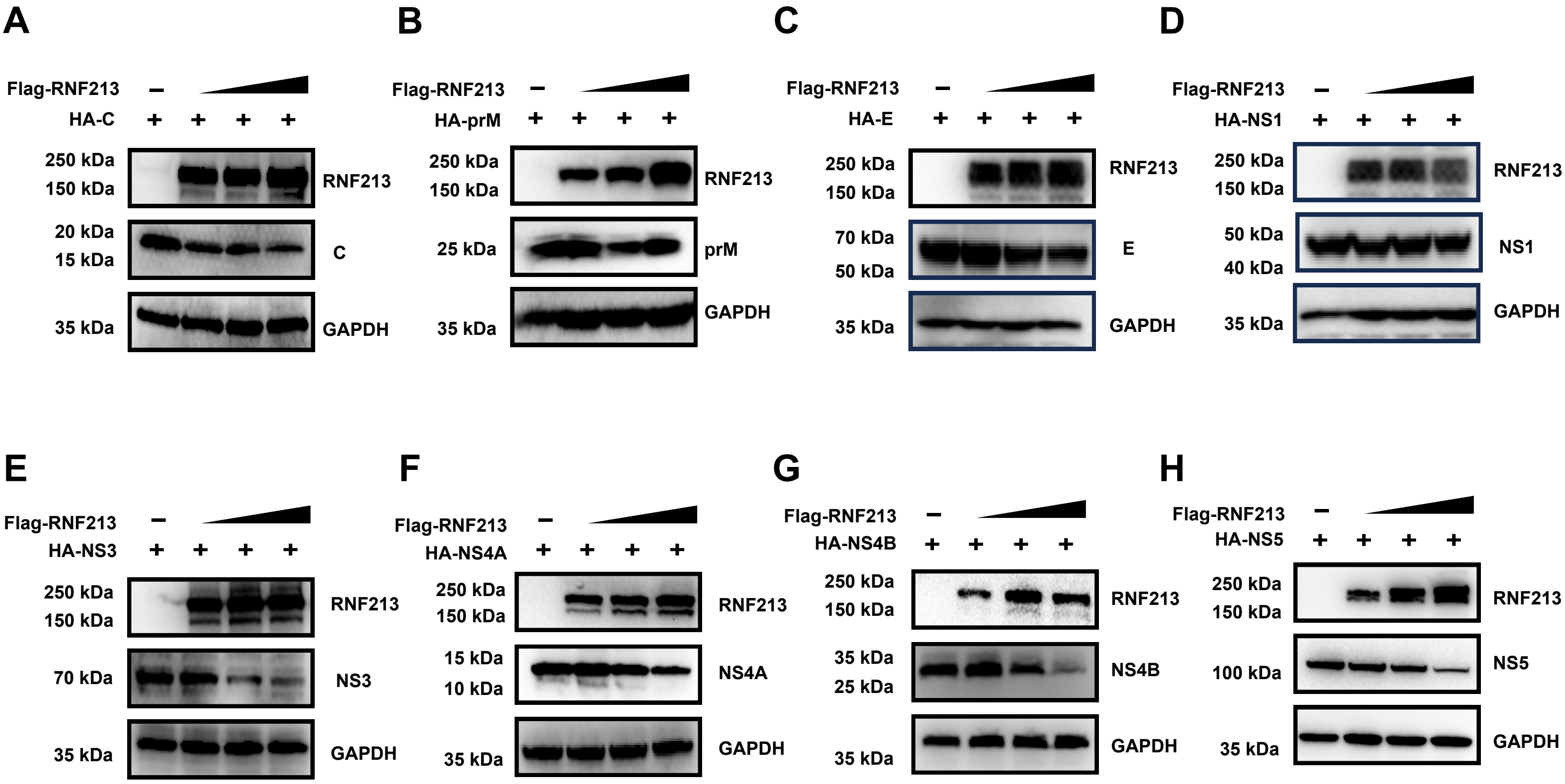

Figure S3. RNF213 degrades ZIKV proteins in a dose-dependent manner.

(A-H) HEK293T cells were transfected with the indicated amount of plasmids encoding Flag-tagged RNF213, together with plasmids encoding HA-tagged ZIKV C, prM, E, NS1, NS3, NS4A, NS4B or NS5 protein. Cell lysates were collected for Western blot analysis. GAPDH served as an internal control.

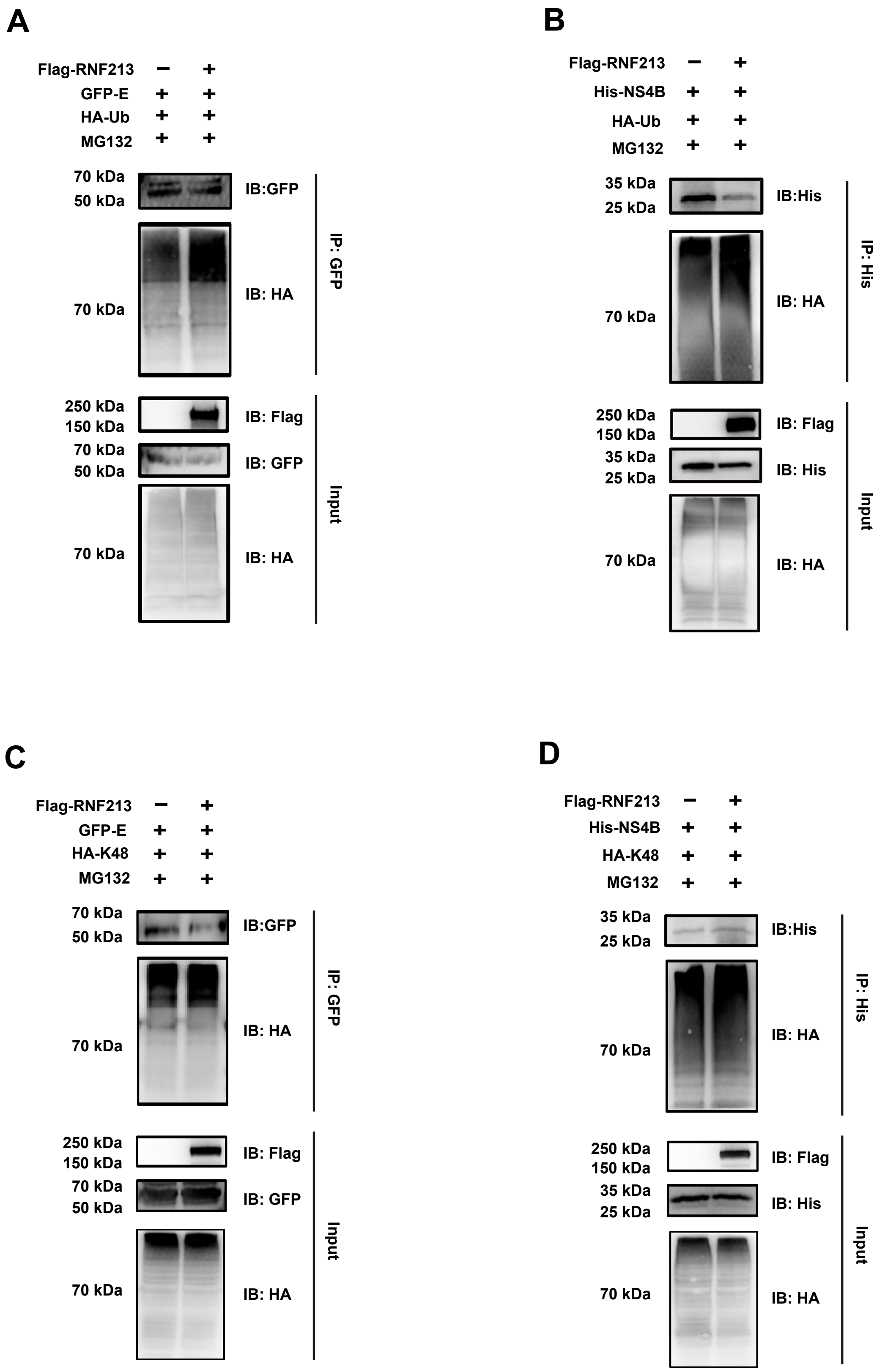

Figure S4. RNF213 promotes ZIKV E and NS4B protein ubiquitination.

(A-B) HEK293T cells were co-transfected with HA-tagged ubiquitin, Flag-tagged RNF213 and GFP-tagged E (A) or His-tagged NS4B (B) plasmid and treated with MG132. Cell lysates were then immunoprecipitated (IP) with anti-GFP or His beads, and immunoblotting (IB) analysis was performed with an anti-HA antibody to detect ubiquitin. (C-D) The same experiment was repeated using HA-tagged K48 ubiquitin plasmid to assess the ubiquitination of ZIKV E and NS4B protein.

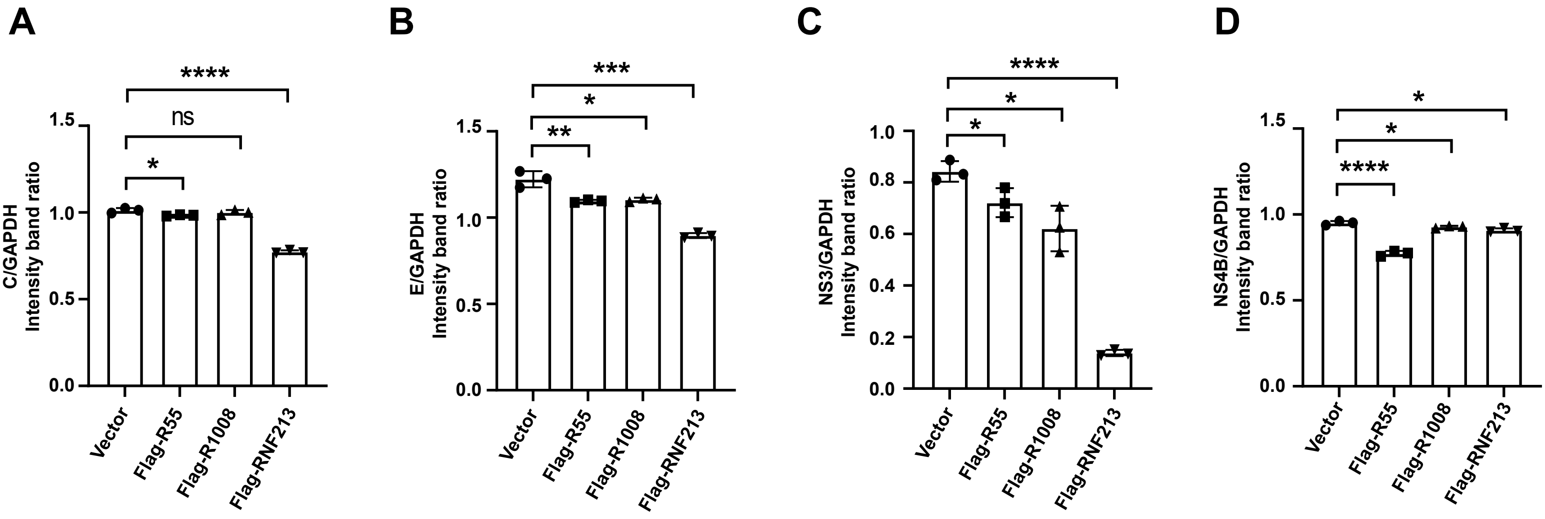

Figure S5. Quantitative analysis of the effects of RNF213 truncation on ZIKV C, E, NS3 and NS4B protein expression.

(A-D). Densitometry analysis of data in Figure 5B-5E. Data are expressed as mean ± SD from three independent experiments (n = 3, ns *P* > 0.05, * *P* < 0.05, ** *P* < 0.005, *** *P* < 0.001, **** *P* < 0.0001).

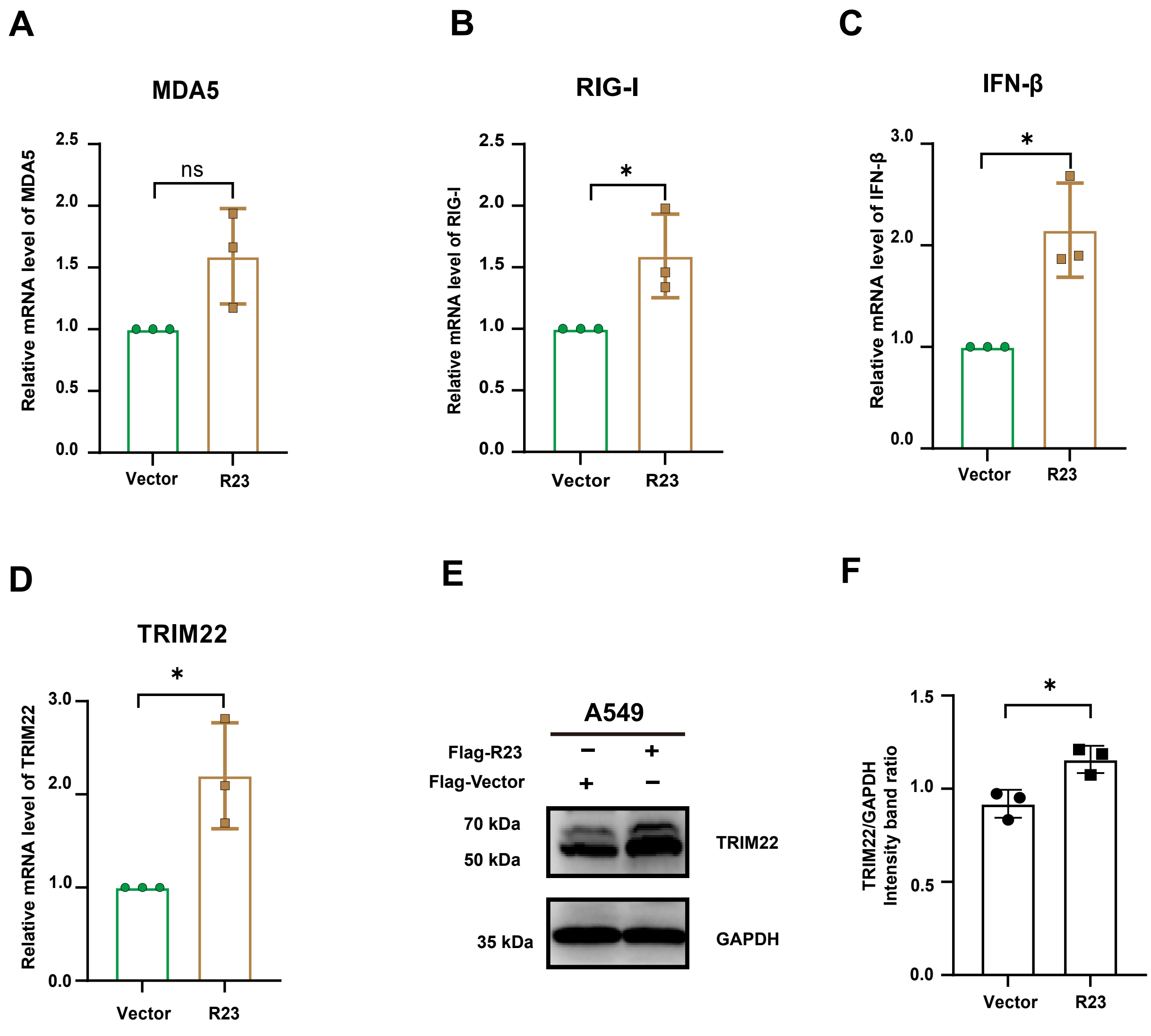

Figure S6. R23 upregulates the RIG-I/MDA5 pathway.

(A-D) A549 cells were transfected with the plasmid encoding Flag-tagged R23 or vector control. After 24 hours, total RNA of cell cultures was extracted and the mRNA levels of *MDA5* (A), *RIG-I* (B), *IFN-β* (C) and *TRIM22* (D) were quantified by RT-qPCR. (E-F) A549 cells were transfected with the indicated amount of plasmid encoding Flag-tagged R23 or vector control. The levels of TRIM22 proteins in A549 cells was measured using Western blot. The protein level of Flag-R23 expressing plasmid was not detected, likely due to its small size. GAPDH served as an internal control. Data are expressed as mean ± SD from three independent experiments (n = 3, ns $P > 0.05$, * $P < 0.05$).

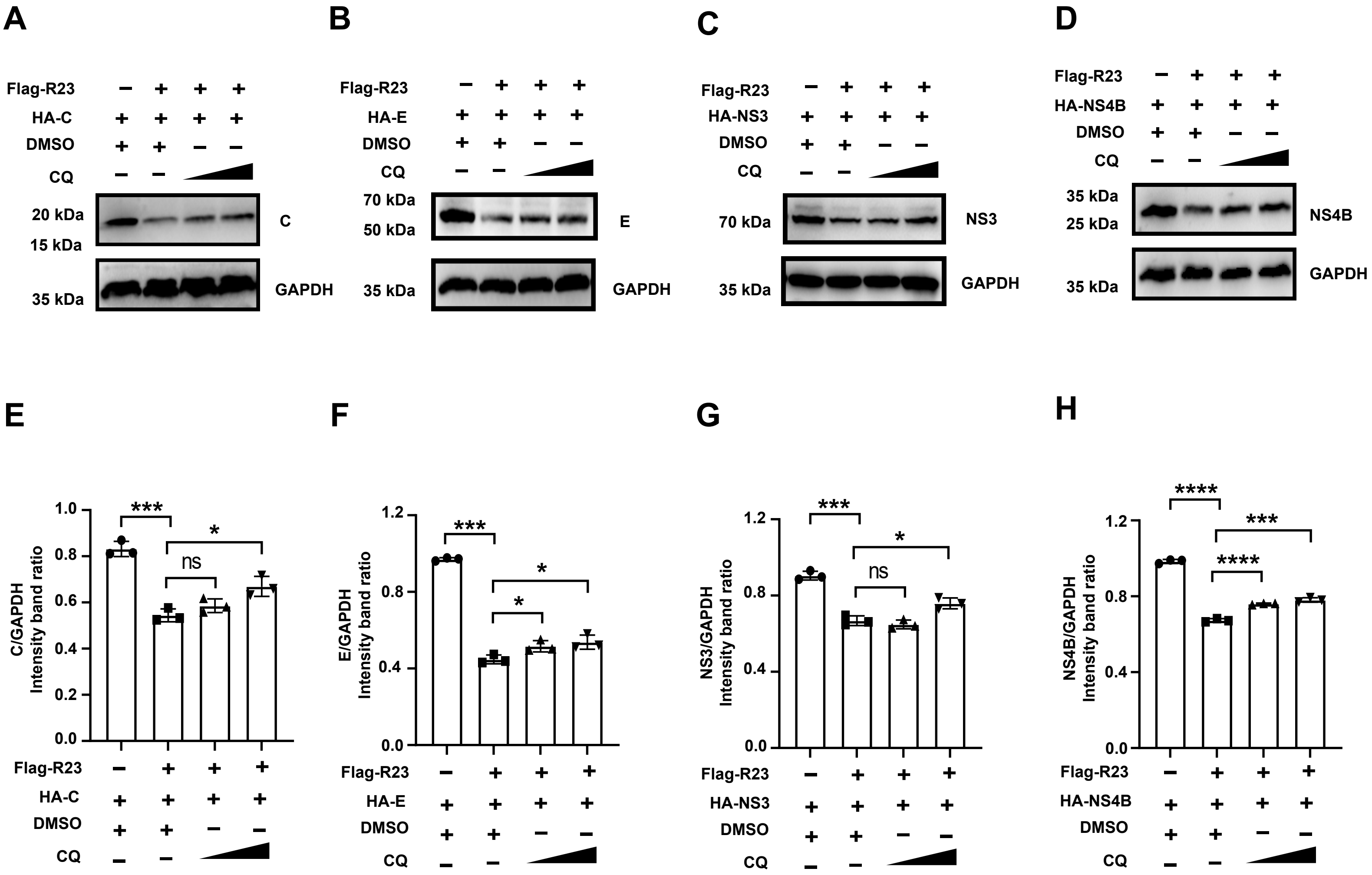

Figure S7. R23 degrades ZIKV C, E, NS3 and NS4B proteins via the lysosomal pathway.

(A-H) HEK293T cells were transfected with plasmid encoding Flag-tagged R23, together with plasmids encoding HA-tagged ZIKV proteins. Chloroquine (CQ) (20 μM and 30 μM) was added at 24 hours post-transfection, and the cells were harvested after 6 hours of incubation, DMSO were used as a negative control. The levels of ZIKV proteins were evaluated by Western blot. The protein level of Flag-R23 expressing plasmid was not detected, likely due to its small size. GAPDH served as an internal control. Data are expressed as mean ± SD from three independent experiments (n = 3, ns $P > 0.05$, * $P < 0.05$, *** $P < 0.001$, **** $P < 0.0001$).

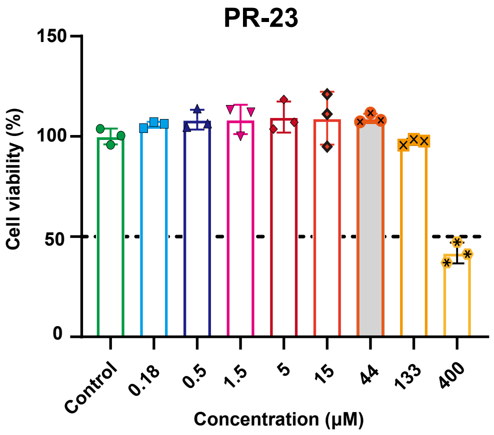

Figure S8. The cytotoxicity of PR-23 in Vero cells.

The cytotoxicity of PR-23 was determined using a CCK-8 assay. Vero cells were treated with the indicated dose of PR-23 or with control for 72 hours, the cells were used to determine the cell toxicity of PR-23 by CCK-8 kit according to the manufacturer's protocols.

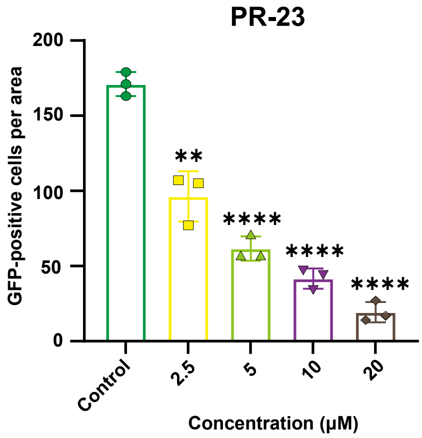

Figure S9. Quantitative measure of the inhibition of PR-23 against ZIKV E protein expression.

Quantitative measure of the inhibition of PR-23 against ZIKV E protein expression was carried out by ImageJ software. GFP-positive cells were counted from three independent areas. Data are expressed as mean ± SD from three independent experiments (n = 3, ** $P < 0.005$, **** $P < 0.0001$).

Gene	Forward Primer	Reverse Primer
GAPDH	GCCTCCTGCACCACCAACTG	ACGCCTGCTTCACCACCTTC
IFN-β	ATGACCAACAAGTGTCTCCTCC	GGAATCCAAGCAAGTTGTAGCTC
MDA5	TCGAATGGGTATTCCACAGACG	GTGGCGACTGTCCTCTGAA
RIG-I	CTGGACCCTACCTACATCCTG	GGCATCCAAAAAGCCACGG
TRIM22	GGTTGAGGGGATCGTCAGTA	TTGGAAACAGATTTTGGCTTC
siRNF213-1#	CCCAGCUAAGGGCUAUGAATT	UUCAUAGCCCUUAGCUGGGTT
siRNF213-2#	GGUCUCAGCUCCUCCGCUATT	UAGCGGAGGAGCUGAGACCTT
siRNF213-3#	GCUCCUCCGCUAACUCAGATT	UCUGAGUUAGCGGAGGAGCTT
siNC	UUCUCCGAACGUGUCACGUTT	ACGUGACACGUUCGGAGAATT
ZIKV	GGTCAGCGTCCTCTCTAATAAACG	GCACCCTAGTGTCCACTTTTTCC
ZIKV probe	FAM-AGCCATGACCGACACCACACCGT-BHQ1	

Table S1. The list of genes primers used in this study.
